# Specific panallergen peptide of *Sorghum* Polcalcin showing IgE response identified based on *in silico* and *in vivo* peptide mapping

**DOI:** 10.1042/BSR20191835

**Published:** 2019-11-15

**Authors:** Chandra Sekhar Bokka, Ganesh Kumar Veeramachaneni, V.B.S.C. Thunuguntla, Naresh Kumar Manda, Jayakumar Singh Bondili

**Affiliations:** 1 Department of Biotechnology, Koneru Lakshmaiah Education Foundation, Green Fields, Vaddeswaram, Guntur Dist. 522 502, Andhra Pradesh, India; 2 Department of Health and Biomedicine, VU Research, Victoria University, Melbourne 3030, Victoria, Australia; 3 Department of Biochemistry, School of Life Sciences, University of Hyderabad, Prof. C.R. Rao Road, Gachibowli, Hyderabad 500 046, Telangana State, India

**Keywords:** Modeling, Peptide Mapping, Polcalcin, Pollen allergen, Sorghum, Th1 and Th2 Cytokines

## Abstract

In India, *Sorghum* plant allergenicity was reported to be approximately 54.9%. *Sorghum bicolor* Polcalcin (Sorb PC) was identified as the panallergen but the specificity of this allergen is yet to be characterized. The present study was aimed to characterize the antigenic determinants of Sorb PC that are responsible for eliciting the IgE response. *In silico* modeling, simulation studies and docking of Sorb PC peptides (PC1–11) against IgG and IgE followed by *in vivo* evaluation was adopted. Peptide docking studies revealed PC 6 with highest G-score −12.85 against IgE followed by PC-11, 5, 1 and 7 (−10.91) peptides. The mice sensitized with PC7 peptide showed interleukin (IL) 4 (IL-4), IL-5, IL-12, TNF-α and GMCSF levels increased when compared with other peptides and controls, signifying a strong T helper type 2 (Th2)-based response. In tandem, the T helper type 1 (Th1) pathway was inhibited by low levels of cytokine IL-2, interferon γ (IFN-γ) and increased IL-10 levels justifying the role of PC7 in allergic IgE response. Considering the above data of overlapping peptides of PC6 and PC7, N-terminal part of the PC7 peptide (*DEVQRMM*) is found to play a crucial role in *Sorghum* Polcalcin allergenic response.

## Introduction

The pathogenesis of respiratory allergic diseases, mainly asthma and rhinitis are generally challenged by aeroallergens. Worldwide, 30% of the population suffer from different types of allergies and has turned out to be an important health concern [1]. A recent survey showed the occurrence of allergic disorders in India at an alarming rate and greater than 25% of the Indian population are suffering from various forms of allergy [2]. The pollen grains from trees, weeds and grasses, foods, fungal spores, insects and dust mites are the major causative agents of allergic reactions [3]. D’Amato et al. [4] had shown that the variance of airborne pollen allergens depends on different climatic conditions, variations in the diurnal and seasonal prevalence. The connection between allergic sensitization, allergen exposure and clinical allergic cross-reactions are vital in elucidating the role of pollens in allergenicity.

Pollen grains contain a mixture of both major and minor (panallergen) allergens; which differ in their level of allergenicity. In our previous work, *Sorghum bicolor* polcalcin (Sorb PC) with an allergenicity score of 0.879 was reported based on *in silico* Algpred screening of known allergenic polcalcin sequences. The Sorb PC gene was identified based on homology [5]. Polcalcin, one of the small acidic, panallergen proteins is ubiquitous in nature and belongs to the Calcium Binding Protein (CBP) family. It shares a common domain termed as EF-hand. Based on the calcium-binding EF-hand motifs (helix–loop–helix) number, three types of polcalcins have been identified. Aln g 4, Amb a 9, Art v 5, Bet v 4, Che a 3, Cyn d 7, Fra e 3, Ole e 3, Phl p 7, and Syr v 3 were found with two domains, Amb a 10 and Bet v 3 with three domains, and Jun o 4 and Ole e 8 with four domains [6]. Functionally, polcalcin is involved in neuronal exocytosis, signal processing and pollen tube growth. Though polcalcins were reported as minor allergens, 10–40% of allergic patients show a high percentage of specific IgE. Polcalcin is vastly conserved among species and their amino acid sequence share a high degree of identity ranging from 60 to 90% with their counterparts from other allergenic sources. As a result, cross-reactivity was observed to be high among the members of the same family [7]. The prevalence of the polcalcin allergen sensitization is dependent on the geographical factors and the level of exposure to this allergen. Polcalcin allergenicity is known, but neither the structure nor the antigenic epitopes of the protein are characterized yet.

Cytokines play a significant role in allergic pathogenesis and inflammation. These are differentiated into pro- (TNF-α, interferon γ (IFN-γ), interleukin (IL) 12 (IL-12) and GMCSF) and anti-inflammatory (IL-4, IL-10) based on the inflammatory switching mechanisms [8]. It is necessary to understand the mechanism of cytokines, which drives the allergic reaction and helps in the development of more effective strategies for the treatment of allergic diseases. T helper type 1 (Th1) and T helper type 2 (Th2) cytokines such as IL-4, IL-5 and GM-CSF along with, TNF-α play a key role in allergen-induced airway leukocyte recruitment [9–11]. Allergen activation directs Th cells belonging to the Th2 subset produces elevated amounts of IL-4, which induce the immunoglobulin class switch to IgE in B cells and is considered an important precondition for an allergic sensitization. Added, IL-4 and other Th2 cytokines contribute to the growth and differentiation of the effector cells involved in allergic and inflammatory reactions. As a result, understanding of the T-cell epitopes of allergen and the cytokine production profiles of allergen specific T cells has become essential for the screening of allergy therapeutics and diagnostics [7].

The effective approach to diagnosis, treatment and prevention of allergy lies in understanding the detailed information about pathogenesis, allergen structure and IgE recognition sites involved in allergenicity. The present study aims at elucidation of the 3d structure of *Sorghum* Polcalcin and identification of peptides responsible for the development of allergenicity using both computational and experimental approaches.

## Materials and methods

### Homology modeling, evaluation and refinement

The *Sorghum* polcalcin 3d structure elucidation was carried out using the Prime homology modeling application. The Polcalcin sequence was retrieved from NCBI (Accession: KC427126, GenBank: AGN33440.1). Application tool inbuilt softwares like BLAST tool, SSPro and PsiPred tools [12] were used in selecting the template and for the prediction of protein secondary structure. Considering the BLAST results, Protein Data Bank (PDB) structure (ID: 1K9U, *Phleum pratense*) was selected as a template for building *Sorghum* polcalcin model having a sequence similarity of 94%. Built model validation was carried using ERRAT [13] and PROCHECK [14] online programs. The built model refinement was done using Desmond molecular dynamic simulations by setting the initial parameters like water model to simple point charge (SPC), orthorhombic periodic box, neutralizing the system by adding a salt concentration of 0.15 M and minimizing the system by setting the iterations to 2000 under convergence threshold of 1.0 (kcal/mol/Å). Further, MDS studies were carried out with a periodic boundary condition in the number of atoms, pressure, temperature (NPT) ensemble, temperature at 300 K, pressure as 1 bar and finally relaxed using the default relaxation protocol integrated in the Desmond. The built model simulations were carried for a period of 100 ns under OPLS force field-2005 and the deviations and fluctuations in the model were analyzed using the trajectory files.

### *Sorghum* polcalcin-Ab and peptide-Ab docking studies

Polcalcin protein-Ab docking studies were carried out to identify the binding site residues of the polcalcin with IgE antibody. Bioluminate module [15] set with default parameters was used to perform this. The interaction profiles of the designed peptides against IgE antibody were also analyzed using the Glide SP-Peptide docking application of Schrodinger suite. Prior to docking studies various steps like protein preparation [16], receptor grid generation and peptide confirmations generation were involved.

### Animal maintenance

Female BALB/c mice, 6 weeks old were bred, maintained in a conventional animal room with 12-h dark/light cycles and were supplemented with pelleted pathogen-free food and water. Ethical Committee for Animal Experiments approved the experimental procedure (CPCSEA - 1242/bc/08). The entire animal work was carried out at Gentox Animal Facilty, Hyderabad.

### Peptide immunization

Individual peptide per group of (*n*=6) female BALB/c mice were sensitized by intra-peritoneal (I.P) injection containing 100 μg peptide adsorbed to the 100 μl Alum (Imject Alum, Pierce Biotechnology, U.S.A.) on 0, 7th and 14th days. Blood samples were collected by retro-orbital bleeding on 21st and 35th days [17]. The collected blood samples were left for 30 min at room temperature to coagulate and then processed further with centrifugation for 10 min at 1000×***g***. After separation, serum was stored at −20°C until used for analysis.

### Overlapping peptide mapping and synthesis

The 14-mer amino acid peptides were designed with an overlapping of seven amino acids using overlapping peptide fragment library software (Sigma–Aldrich). The designed peptides were commercially synthesized on Fmoc system (JPT Peptide Technologies GmbH, Berlin, Germany). All the peptides were supplied in the form of lyophilized powder with a purity of >95% analyzed by ultra HPLC and LC-MS/MALDI-MS.

### Cytokine assays

The serum cytokine profiling was performed using a commercially available multiplexed kit, Bio-Plex Pro™ Mouse cytokine Th1/Th2 Assay (Bio-Rad Mouse Multi-Cytokine Detection System; Bio-Rad Laboratories, India). The cytokines assay was performed to measure IL-2, IL-4, IL-5, IL-10, IL-12 (p70), GM-CSF, TNF-α and INF-γ using commercially available Bio-Rad kit. In brief, the mouse serum collected was diluted to 1:5 and processed as per the manufacturer’s protocol. Cytokine concentrations were quantified using Bioplex protein array system (Bio-Rad) following the manufacturer’s instructions. The assay sensitivity was less than 10 pg/ml and ranged from 0.2 to 32000 pg/ml with an inter- and intra-assay CV of less than 10% [18].

### Total IgE and IgG assays

Serum IgE levels in the mouse serum were measured using MILLIPLEX® MAP Mouse IgE single plex magnetic bead Kit (Merck Millipore, India) as per the manufacturer’s protocol. All the samples were measured in duplicates and estimated using Luminex Magpix [19]. The total IgG concentration in mouse serum was estimated using (Invitrogen, India) ELISA kit as per the manufacturer’s protocol. The results obtained were means of duplicate determination with variation less than 10%. Further, the IgE and IgG concentrations were determined by comparing the mean OD values of the tested sera with the mean OD values of the standard. IgE and IgG titers were calculated by multiplying the dilution factor of the test sera and expressed in ng/ml.

### Statistical analysis

To calculate the statistical functions, GraphPad Prism v.7 analysis program was used. The median of the parameter, standard deviation (SD) and the arithmetic mean were calculated. The non-parametric *t* test: two-sample assuming unequal variance was applied to compare measurable characteristics between the groups. The differences were considered significant at *P*<0.05.

## Results

### Homology modeling and molecular dynamic simulations

The Polcalcin 3d structure was developed following the Prime homology modeling protocol. Energy-based model was built and validated with Verify 3d and ERRAT. Verify 3d, a Ramachandran plot developer tool reported 90.3% of amino acids in most favored regions and the remaining 9.7% amino acids were found in additionally allowed regions. ERRAT tool generated overall quality factor considering the data of non-bonded atomic interactions present in the built structure. The built model score was found to be 84.72. ERRAT score greater than 90 was considered better for models and hence the built model required further refinement to enhance the model quality. Consequently, to refine the built model, molecular dynamics study was carried out for a period of 100 ns.

**Figure 1:**
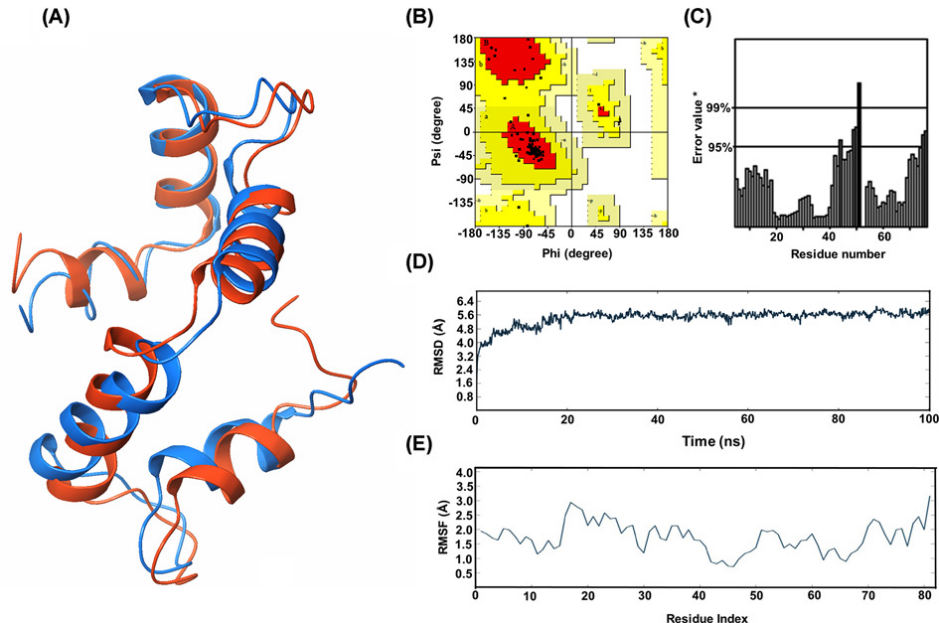
Homology modeling and molecular dynamic simulation results of Sorghum polcalcin (**A**) Homology model of *Sorghum* polcalcin (Blue color model: before simulations and Orange colored: after simulations). (**B**) Ramachandran plot of polcalcin (Red: most favored regions; Yellow: additionally allowed regions; Light yellow: generously allowed regions; White: disallowed region). (**C**) ERRAT score of the built model after simulations. (**D**) Deviations graph of polcalcin after simulations. (**E**) Fluctuations graph shown by the polcalcin residues during simulations.

The trajectory file obtained by the end of simulation run was analyzed to calculate the deviation and fluctuations shown by the built model (Figure 1D-E). The overall deviations of the built model were in the range of 3.2–5.8 Å. Inclination in the deviations from 3.2 to 5.6 Å was observed in the initial 0–20 ns frame and from 20 ns, a steady state in the deviations graph was observed at approximately 5.6 Å. Fluctuations made by the amino acids during the simulation run were in between 1.0 and 3.2 Å. The fluctuations range was quite acceptable with majority of the amino acids displaying below 2.5 Å. The highest fluctuation, i.e. 3.2 Å was shown by the tail end amino acid present at 3′ end of the sequence. In general, the tail end regions fluctuate more during simulations to attain a stable conformational state (Figure 1A). To validate the simulated model, RMSD-based cluster center frame obtained from the trajectory analysis was selected and evaluated using Ramachandran plot and ERRAT score to check for the improvement in the model scores. The ERRAT score of the refined model was increased from 84.72 to 91.42 indicating that the model underwent refinement during the simulations run, justifying the increase in overall modeled protein stability (Figure 1C). Subsequently, changes in the Ramachandran plot were also observed with 87.5% amino acids in the most favorable region and 12.5% amino acids were in allowed regions with no outliners (Figure 1B).

### Docking studies

**Figure 2:**
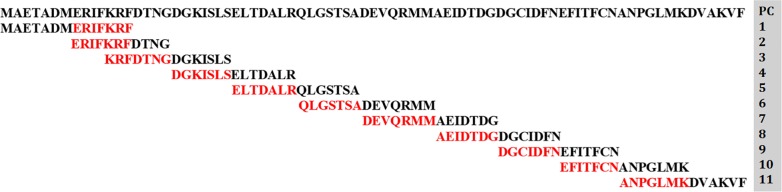
*Sorghum* polcalcin sequence aligned with designed peptides (PC1–11) Each peptide has seven overlapping amino acids at the C-terminal.

To detect the Ag–Ab interacting domains, protein–protein docking study involving IgE antibody and polcalcin was executed. Antibody structure was retrieved from PDB (ID: 2VXQ) [20] and using the protein preparation wizard of maestro, the raw structure was refined. All the simulated trajectory frames of the modeled polcalcin structure were clustered and the cluster center frame having the minimal energy, deviations and fluctuations was adopted for the binding studies. From the docking results, the tail-end sequence of the polcalcin was found to produce interactions with the paratope region of the IgE antibody. The present study could not elucidate the overall polcalcin amino acids involved in establishing interactions with the IgE paratope region. Simultaneously, the relative surface accessibility study of the polcalcin protein was also undertaken using online tool NetSurfP-2.0 (http://www.cbs.dtu.dk/services/NetSurfP/), which highlights the amino acid residues accessibility nature, i.e. buried or exposed. Majority of the buried residues were found in the helices (Supplementary Figure S1) and hence were not accessible to interact with IgE during the *in silico* binding studies. Consequently, to study in detail the overall polcalcin amino acid interactions with IgE, the polcalcin sequence was fragmented into 11 peptides (Figure 2) using overlapping peptide fragment library software and their binding modes with the IgE paratope region were analyzed. Along with IgE, IgG antibody binding study was also adopted for comparative analysis and also to affirm the specificity of peptides. The IgG antibody was imported from the PDB (ID: 4J4P) [21] into the maestro and further processed using the same procedures that were implied to the IgE antibody.

**Figure 3:**
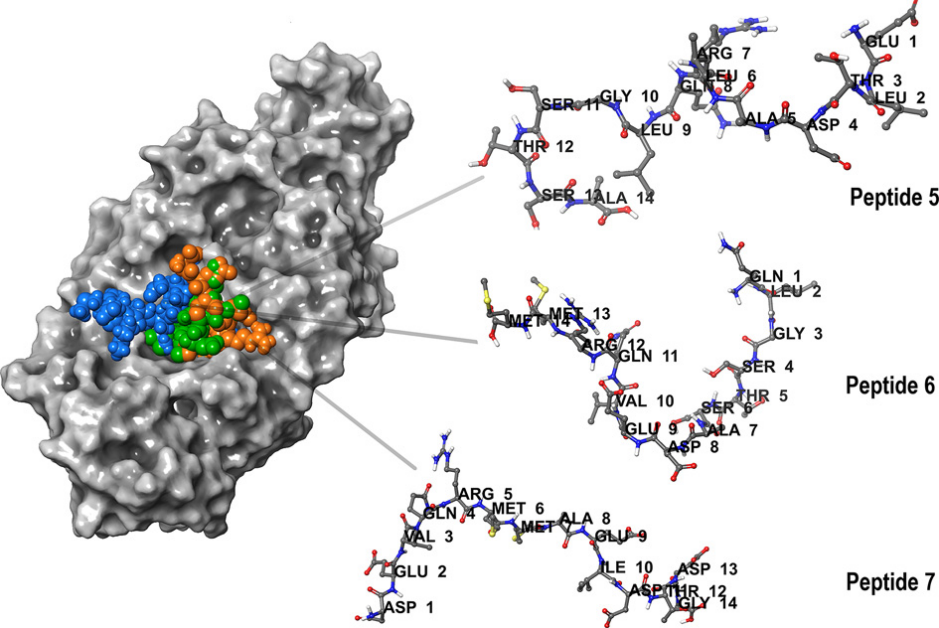
*Sorghum* polcalcin peptides PC5 (green colored), PC6 (blue colored) and PC7 (orange colored) displaying binding modes within the paratope region of IgE antibody after peptide docking studies Amino acid interactions of the peptide are highlighted.

**Figure 4:**
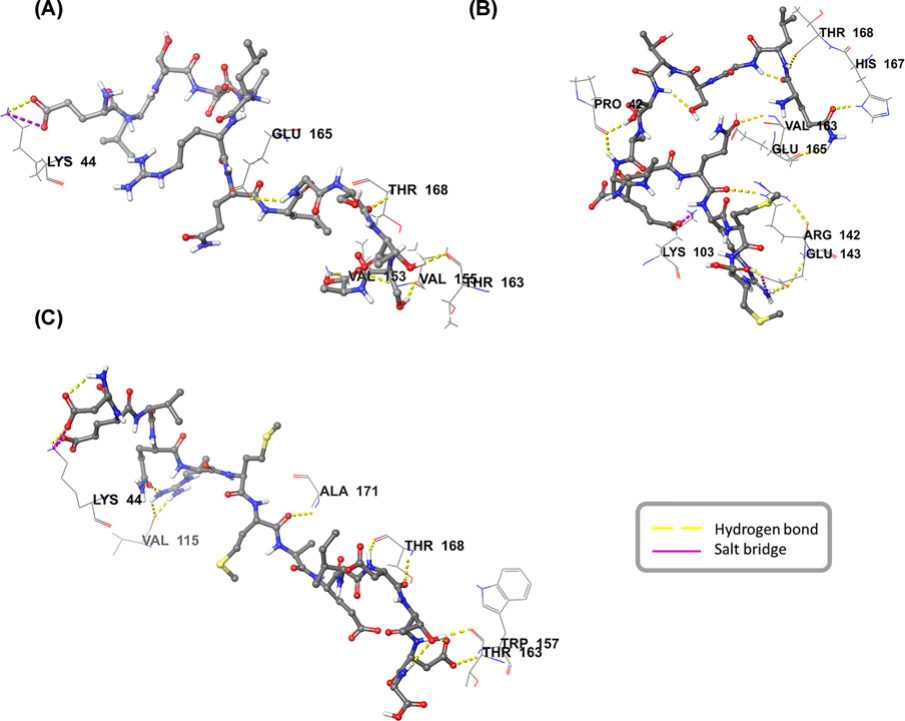
Molecular interactions of polcalcin peptides with IgE H-bond and salt bridge interactions between the *Sorghum* polcalcin peptides and IgE antibody amino acids: (**A**) Peptide PC5, (**B**) Peptide PC6, (**C**) Peptide PC7 interactions.

The mode of interactions between the peptide and antibody was analyzed and their G-scores were reported (Table 1). Peptide-6 displayed highest score against IgE followed by peptides 11, 5, 1 and 7. Peptide G-scores in complex with IgG were low in comparison with IgE complex. Based on the G-scores, high scoring peptide 6 and its overlapping fragments, peptide 5 and 7 were considered for further study. The binding modes of the three peptides PC5–7 against IgE were displayed in the Figure 3. Peptide 6 and IgE complex were maintained by nine hydrogen bonds (H-bonds) and two salt bridges (Figure 4A). Out of nine H-bonds, four H-bonds interactions were observed with heavy chain amino acids and five H-bonds with light chain amino acids. The two salt bridges made by the peptide were linked to light chain amino acids. Peptide 5 exhibited a total of seven H-bonds and one salt bridge with the paratope region amino acids of IgE (Figure 4B). Among the seven, six H-bonds were with the heavy chain amino acids and one was with light chain amino acid. The salt bridge was formed with the heavy chain amino acid of the IgE antibody. The interaction profile of the peptide 7-IgE complex was sustained through ten H-bonds and two salt bridges (Figure 4C). All the interactions were found with heavy chain amino acids of IgE antibody. The interaction profiles of PC5–7 against IgE were reported in the Table 2.

**Table 1:** Docking G-scores of the *Sorghum* Polcalcin peptides PC1–11 against IgE and IgG antibody obtained from peptides docking studies

PC No.	Peptide sequence	G-score	
		IgE	IgG
1	MAETADMERIFKRF	−11.12	___
2	ERIFKRFDTNG	−10.45	−8.78
3	KRFDTNGDGKISLS	−10.85	−8.64
4	DGKISLSELTDALR	−10.67	−7.46
5	**ELTDALRQLGSTSA**	−11.42	−8.94
6	**QLGSTSADEVQRMM**	−12.85	−8.90
7	*DEVQRMM*AEIDTDG	−10.91	−8.02
8	AEIDTDGDGCIDFN	−9.62	−7.18
9	DGCIDFNEFITFCN	−9.79	___
10	EFITFCNANPGLMK	−9.91	−3.91
11	ANPGLMKDVAKVF	−11.79	−8.12

Highest scoring peptide PC6 and its overlapping amino acids of PC5 and PC7 were highlighted in bold.

**Table 2:** Interaction profiles of the selected three peptides PC5-7, bold highlighted amino acids showed interactions with the paratope region amino acids of the IgE

Peptide	IgE Paratope region amino acid interactions	
	H-bond	Salt bridge
ELTDALRQLGSTSA (PC5)	E: Lys 44(H)	L:Lys 44(H)
	G: Glu 165(L)	
	S: Thr 168(H)	
	T: Thr 163 (H)	
	S: Val 155(H)	
	A: Val 153 (2H-bonds-H) and Val 155 (H)	
QLGSTSADEVQRMM (PC6)	Q: Glu 165 (L) and His 167 (H)	E:Lys 103 (L)
	L: Thr 168 (H)	R:Glu 143 (L)
	S and D: Pro 42 (2 H-bonds-H)	
	Q: Val 163(L) and Arg 142(L)	
	R: Glu 143 (2 H-bonds-L)	
*DEVQRMM*AEIDTDG (PC7)	D and E: Lys 44 (H)	D: Lys 44(H)
	R: Val 115 (2 H-bonds-H)	E: Lys 44(H)
	M: Ala 171(H)	
	D: Thr 168 (2 H-bonds-H)	
	T: Thr 163(H)	
	D: Trp 157 (H)	

Abbreviations: (H), heavy chain; (L), light chain.

### Cytokine studies

**Figure 5:**
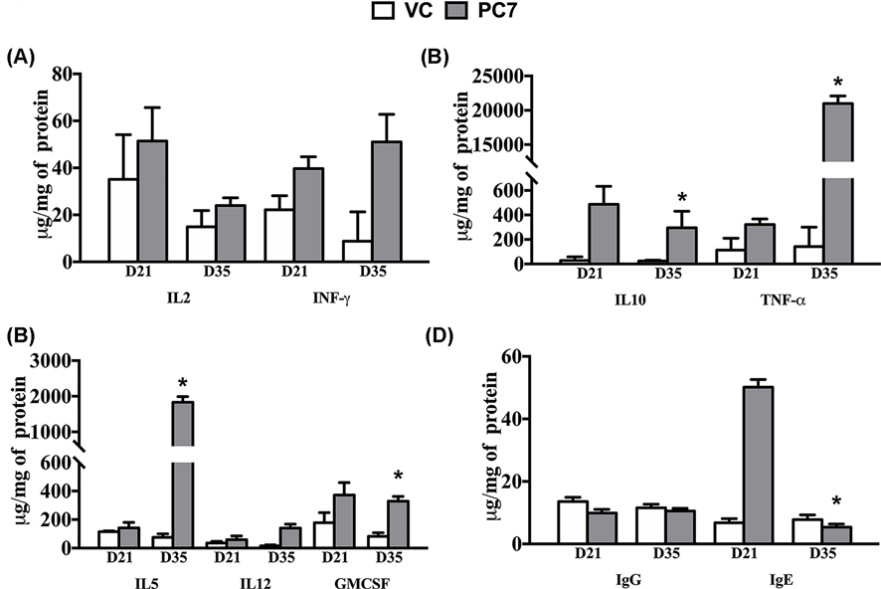
Cytokines profiles of polcalcin peptide 7(PC-7) Quantification of cytokines: (VC: Vehicle Control; PC-7: Polcalcin peptide-7) PC-7 peptide sensitized mouse serum samples were estimated for (**A**) IL2 and INFγ, (**B**) IL10 and TNF-α, (**C**) IL5, IL12 and GMCSF, (**D**) IL4, (**E**) IgG values were low when compared with VC and whereas IgE was significantly increased on 21st day. The data shown are Mean ± SD (x: represents significance between VC and peptide on Day 21; y: represents significance between VC and peptide on Day 35; *: represents significance between Days 21 and /35 of the same peptide).

To elucidate the mechanisms adopted in eliciting the allergic reaction by *Sorghum* Polcalcin, we examined cytokine levels in serum of mice stimulated with PC1-9 peptides *in vivo* Peptide 7 showed a strong Th2-based response with significantly increased levels of IL-4, IL-5, IL-12, TNF-α and GMCSF when compared with non-sensitized mice, vehicle control and other peptides (Figure 5). The total cytokine concentration of IL-4, IL-5, IL-12, TNF-α, IFN-γ and GMCSF were found to be increased on 21^st^ day compared with vehicle control. However, on the 35th day, IL-5, IL-12 and TNF-α showed significant increase while GMCSF concentration was maintained almost at the same level.

Concurrently, IL-2, the Th1 pathway cytokine level was though found elevated on the 21st day, gradually decreased when compared with vehicle control by 35th day. Overall, the Th1 pathway cytokines (IL-2 and IFN-γ fold change) remained at low levels by the 35th day, thereby promoting the production of IgE (Figure 5). The IL-10 concentration was also found to be raised significantly on the 21st day but decreased by 35th day (Figure 5). There was no significant difference found in mice sera with other peptides (Supplementary Figures S2 and S3) injected when compared with controls.

### Total IgE and IgG study

Higher serum total IgE levels were detected in the peptide PC7 (50.16 μg/mg of protein) sensitized mice when compared with vehicle control (6.77 μg/mg of protein) and other peptides on 21st day. The levels were significantly decreased for the peptide PC7 (*P*=0.0008) to 5.35 μg/mg of protein, respectively on the 35th day (Figure 5). Concomitantly, the total IgG concentration was almost at the same level and was found to be 9.87 μg/mg of protein on the 21st day and 10.56 μg/mg of protein on the 35th day, respectively. Other peptides IgE and IgG levels were very low and had no significance when compared with PC7 (Supplementary Figure S4).

## Discussion

To study the epitopic regions of polcalcin, a three-dimensional structure was built due to the unavailability of crystal structure in the PDB. The built model was evaluated and based on the scores, the model was further refined to get a stable conformational state of the polcalcin. Deviations reported during the simulation run were above the range because of the presence of loop regions in the structure. Generally, for the small and globular proteins, the acceptable RMSD was 1.0–3.0 Å. Beyond this range, it indicates that the protein was undergoing a lot of conformational changes during the simulations run for attaining a stable conformational state. Based on deviations reported, it was inherent that the model underwent vast conformational changes within 20 ns and from there on the model attained stability in the backbone and continued with small conformational changes in the side chains. Fluctuations made by the model during the simulation run were within the acceptable range. An increment in the ERRAT score was observed after the refinement, specifying the refinement of the model, i.e. before and after simulations. Ramachandran plot after simulations was limited to only favorable and allowed regions without any outliners. This implies that the changes made by the amino acids during the simulation run helped the model to attain a more stable conformational state.

Prior to the peptide docking studies, protein–protein docking study was performed to identify the interactions between the IgE paratope amino acids and the polcalcin. The docking approach revealed how the polcalcin binding to the antibody was limited only to the exterior amino acids of the IgE antibody and hence peptide mapping approach was adopted. Among the 11 peptides compared against IgE, peptide 6 showed high score but peptide 7 had more number of H-bond interactions with IgE paratope region than peptides 5 and 6.

Antigen Presenting Cells (APCs) initiate Type-I allergy pathogenesis by phagocytosis of allergens and thereby presenting antigen to naive T cells. Typically, of the four different populations of CD4^+^ T cells, the Th1, Th2, T helper 17 (Th17) and regulatory T (Treg) cells [22], the Th2 cells mostly produce IL-4 and IL-5, which promote IgE synthesis. Contrasting with Th2 cells response, the Th1 cells are involved in down-regulating IgE production by releasing cytokines IL-2 and IFN-γ [23]. The balance between Th1- and Th2-pathways is considered as the foremost important phase in immune homeostasis and also in allergic disease [24]. Treg cells are majorly involved in regulation of the Th1–Th2 equilibrium and suppression of the allergic response [25]. Cytokine production is crucial in both the early and late phases of the allergic response. In the course of the early phase reaction, allergen re-exposure activates the release of cytokines IL-3, IL-4, IL-9 and IL-13, responsible for immediate hypersensitivity. In the late phase allergic reaction, Th2 cells and mast cell-derived cytokines namely IL-3, IL-5 and GM-CSF are involved in the eosinophil activation and recruitment of leukocytes to the region of allergen exposure. Whereas in excessive inflammation and airway remodeling conditions, cytokines like IL-5, IL-9, IL-13 and TNF activity plays an important role in the late-phase asthmatic reactions [26,27]. The cytokine assay results confirm that the mouse sensitized with PC7 peptide was only proactive in promoting the Th2 cells by elevating the levels of IL5, IL12 and TNF-α on the 35th day, significantly when compared with the 21st day. Concomitantly, GMCSF the other Th2 promoter was found maintaining same concentrations on both 21st and 35th days. In tandem, the Th1 cell promoters IL-2 concentration was found decreased (35th day) when compared with 21st day. Only PC7 peptide produced significant response toward Th2 cells. No comparative cytokine profiles are reported for any of the polcalcins identified till date. In addition, the peptide PC7 sensitized mouse also showed elevated levels of serum IgE levels on 21st day compared with the control and further decrease in serum concentrations were observed on 35th day in line with the IL-4 levels which was also found elevated only on day 21 promoting IgE production and onset of Th2 pathway. Unlike IgE, IgG concentrations in the PC7 sensitized mouse serum remained almost same on both 21st and 35th days in line with low production of IL-2 when compared with VC than IL-4 levels which favored the triggering of the Th2 pathway.

IL-5 levels were found justified on 35th day which is a late phase allergic cytokine identified as major maturation and differentiation factor for eosinophils [28]. Though IFN-γ was found on both 21st and 35th days, the fold change was too low when compared with IL-4 and could be attributed to the suppression of Th-1 pathway along with anti-inflammatory cytokine IL-10 which promotes favorable modulation of IgG_4_ to IgE ratio [29]. High levels of TNF-α on 35th day imply its major role in the pathogenesis of allergy and its contribution to allergy development both in the early and late stages. Allergen exposure leads to the production of TNF by sensitized macrophages, mast cells, dendritic cells. It also promotes Th2 responses, resulting in high levels of IL-4, IL-5 and IL-13, which further activate eosinophils, mast cells and basophils. Further, TNF is both induced and secreted by Th2 cells promoting the production of IgE isoforms from B cells which are antigen-specific [30].

Based on the above computational and animal studies, only PC7 peptide was found responsible for the allergenic response following Th2 pathway. Despite the fact that docking studies revealed three peptides PC5, 6 and 7 to be more significant with respect to binding modes against IgE and IgG, only PC7 peptide showed augmented Th2-based cytokine response in mice and also exhibited higher titers of IgE in mice sera when compared with other peptides. The PC7 peptide showed more H-bond interaction when compared with peptide 6 and in specific the N-terminal part of the PC7 peptide (*DEVQRMM*) represented five H-bonds and two salt bridge interactions. Through peptide mapping and docking studies, the specific binding domain of the polcalcin was determined and the activity of the determined peptide was validated through *in vivo* studies. The combination of computational and experimental approach made the present study more specific in determining the antigenic domain of the Polcalcin triggering IgE response.

## Conclusions

In conclusion, the present study highlights the 3d modeling of *Sorghum* Polcalcin and based on *in silico* and *in vivo* data of peptides screened the N-terminal part of the PC7 peptide (*DEVQRMM*) is identified to play a vital role in eliciting Th2 allergenic response and thereby leading to the production of IgE. The structural and cytokine data confirm that the PC7 peptide is responsible for allergenic response and could probably be useful in developing the new diagnostic kits targeted toward allergen screening.

## Supplementary Material

Supplementary Figures S1-S4

## Abbreviations

GMCSF: Granulocyte Macrophage Colony Simulating Factor
H-bond: Hydrogen bond
IFN-γ: Interferon γ
IL: Interleukin
PDB: Protein Data Bank
Sorb PC: *Sorghum bicolor* Polcalcin
Th1: T helper type 1
Th2: T helper type 2
Treg: regulatory T
