# Supplementary material for: Specific panallergen peptide of Sorghum Polcalcin showing IgE response identified based on in silico and in vivo peptide mapping
Source: Biosci Rep. 2019 Nov 15;39(11):BSR20191835. doi: 10.1042/BSR20191835 (PMC6859114; doi:10.1042/BSR20191835)
Supplement: Supplementary Figures S1-S4 [file BSR-2019-1835_supp.pdf]

Supplementary data

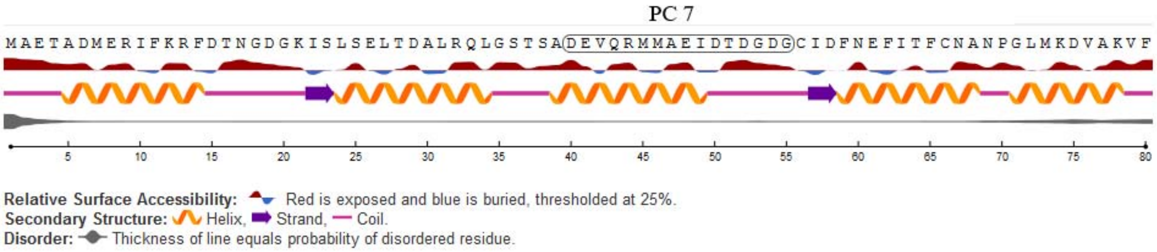

Supplementary Figure 1. Relative solvent exposure analysis of polcalcin residues. Peptide PC7 region is boxed highlighting 5 amino acids buried in and most of the peptide in helix form.

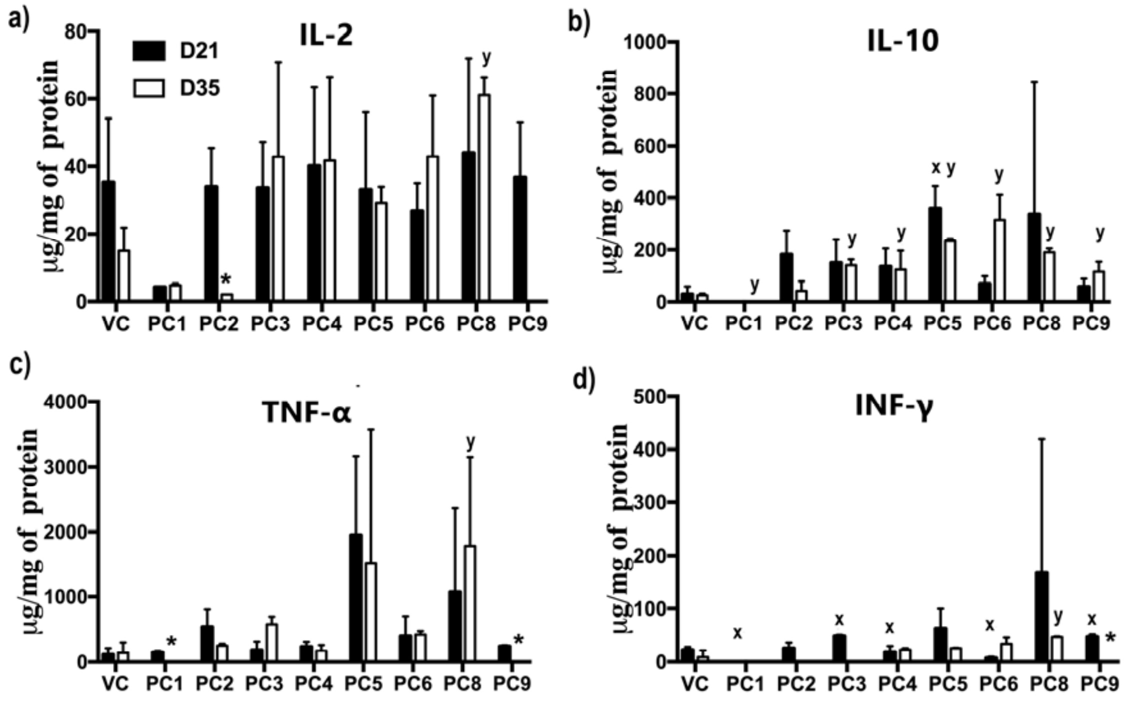

Supplementary Figure 2. Quantification of Cytokines: (VC: Vehicle Control; PC1-9 except PC7; PC: Polcalcin peptide) peptides sensitized mouse serum samples were estimated for (a) IL2 (b) IL10 (c)TNF-α and (d) INFγ (d) IgG. D21 represents 21st day and D35 as 35th day. The data shown is Mean± SD (x: represents significance between VC and peptide on Day 21; y: represents significance between VC and peptide on Day 35; *: represents significance between Day 21 and Day 35 of the same peptide).

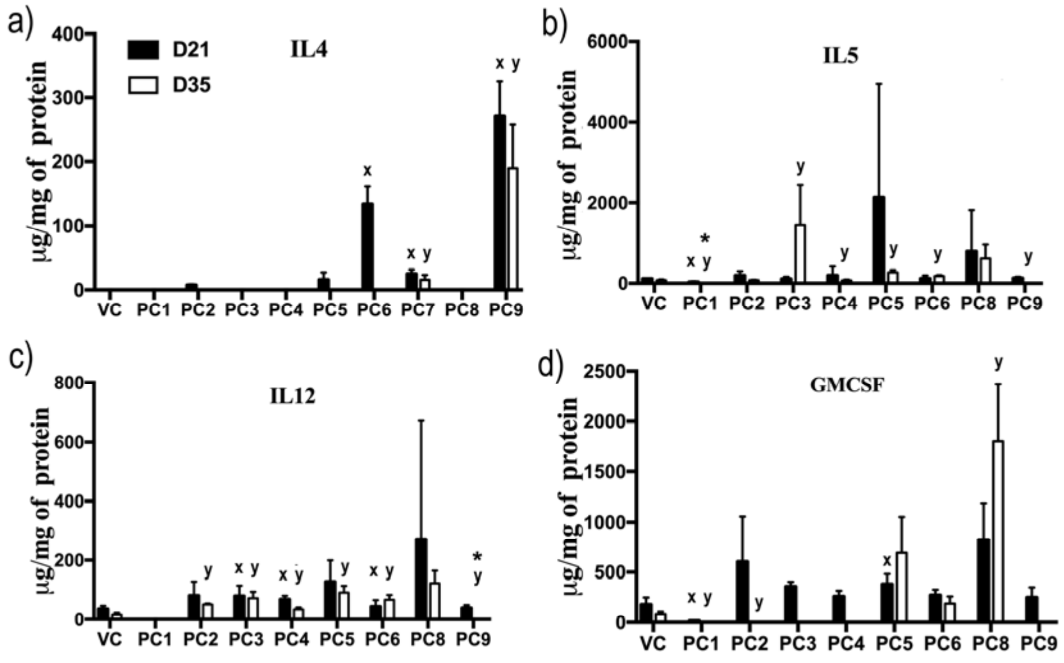

Supplementary Figure 3. Quantification of Cytokines: (VC: Vehicle Control; PC1-9 except PC7; PC: Polcalcin peptide. Peptides sensitized mouse serum samples were estimated for (a) IL4 (b) IL5 (c) IL12 and (d) GMSCF. D21 represents 21st day and D35 as 35th day. The data shown is Mean± SD (x: represents significance between VC and peptide on Day 21; y: represents significance between VC and peptide on Day 35; *: represents significance between Day 21 and Day 35 of the same peptide).

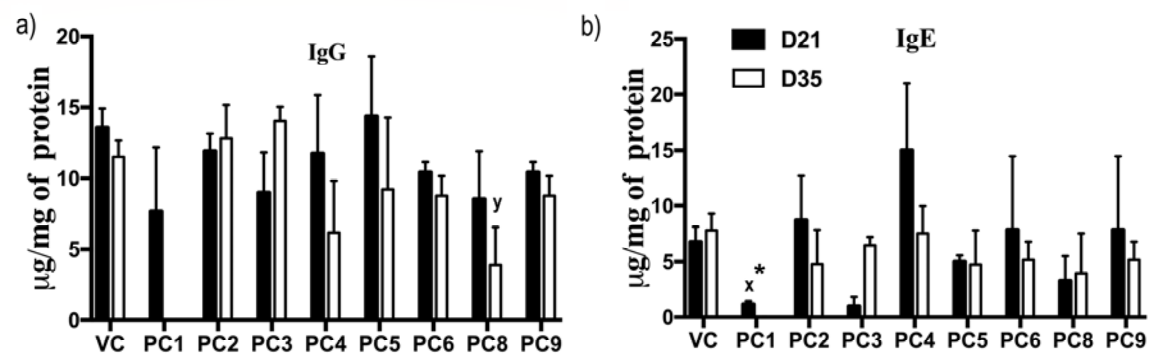

Supplementary Figure 4. Quantification of Cytokines: (VC: Vehicle Control; PC1-9 except PC7; PC: Polcalcin peptide. Peptides sensitized mouse serum samples were estimated for (a) IgG (b) IgE data on 21st day (D21) and 35th day (D35). The data shown is Mean± SD (x: represents significance between VC and peptide on Day 21; y: represents significance between VC and peptide on Day 35; *: represents significance between Day 21 and Day 35 of the same peptide).

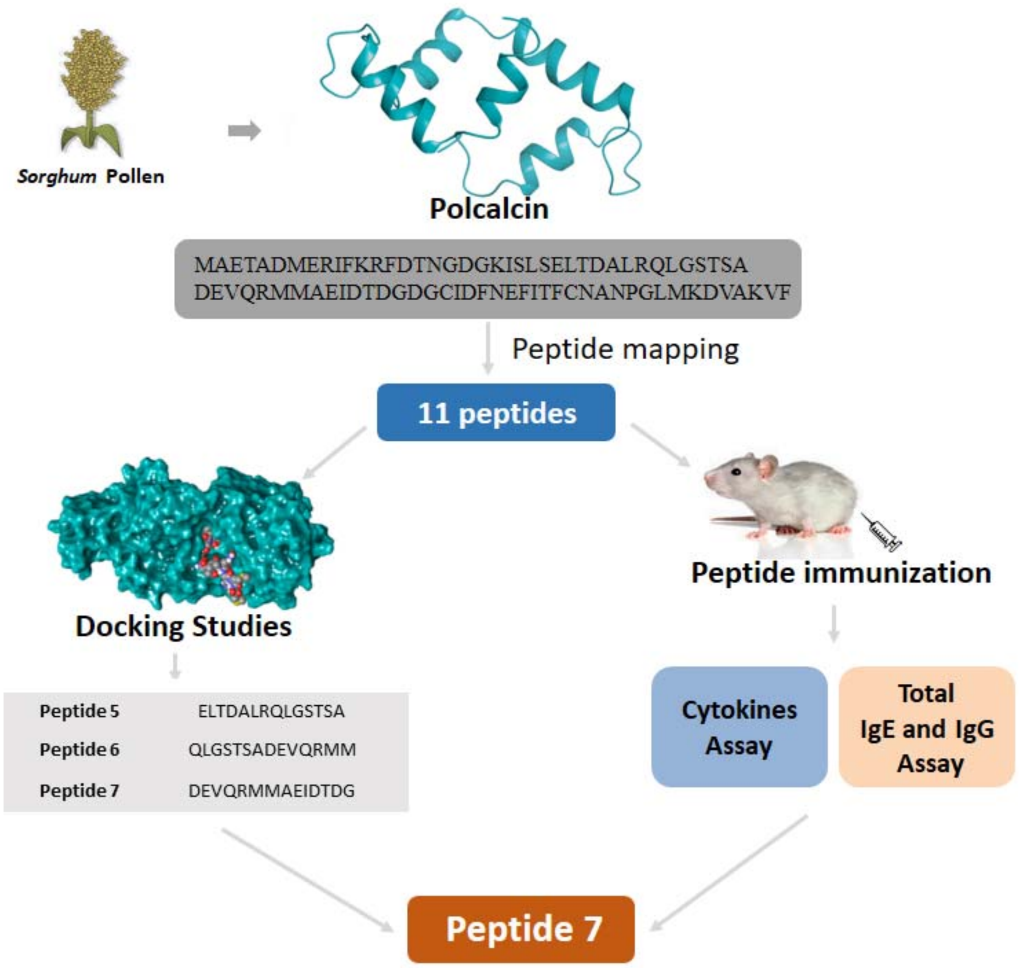

Graphical abstract
